# Self-selected Recovery in High-intensity Interval Training Promotes Positive Responses in Affect, Pleasure, and Mood in Young People

**DOI:** 10.2174/0117450179378796260109054752

**Published:** 2026-02-18

**Authors:** Leandro Sant’Ana, Jeferson Macedo Vianna, Bruno Travassos, Fábio Yuzo Nakamura, Diogo Teixeira, Fabiana Rodrigues Scartoni, Amandio Dias, Raul Antunes, Filipe Rodrigues, Rui Matos, Sérgio Machado, Diogo Monteiro

**Affiliations:** 1 Postgraduate Program in Physical Education, Federal University of Juiz de Fora, Minas Gerais, Brazil; 2 Department of Biophysics and Physiology, Federal University of Juiz de Fora, Juiz de Fora, Brazil; 3 Department of Sports Science, University of Beira Interior, Covilhã, Portugal; 4 Research Center in Sport, Health and Human Development, Vila Real, Portugal; 5 Portugal Football School, Portuguese Football Federation, Cruz Quebrada, Portugal; 6 Research Center in Sports Sciences, Health Sciences, and Human Development, University of Maia, Maia, Portugal; 7 Faculty of Physical Education and Sport, Lusófona University, Lisbon, Portugal; 8 Research Center in Sport, Physical Education, and Exercise and Health (CIDEFES), Lisbon, Portugal; 9 Department of Physical Education, Catholic University of Petropolis, Rio de Janeiro, Brazil; 10 Egas Moniz School of Health and Science, Monte de Caparica, Portugal; 11 Egas Moniz Center for Interdisciplinary Research (CiiEM); Egas Moniz School of Health & Science, Caparica, Almada, Portugal; 12 Integrative Movement and Networking Systems Laboratory (INMOV-NET LAB) - Egas Moniz Center for Interdisciplinary Research (CiiEM), Egas Moniz School of Health & Science, 2829-511, Caparica, Almada, Portugal; 13 Sport Physical Activity and Health Research & Innovation Center (SPRINT), Rio Maior, Portugal; 14 ESECS, Polytechnic University of Leiria, Leiria, Portugal; 15 Research Center in Sports, Health, and Human Development (CIDESD), Covilhã, Portugal; 16 Laboratory of Panic and Respiration, Institute of Psychiatry of Federal University of Rio de Janeiro (IPUB-UFRJ), Rio de Janeiro, Brazil

**Keywords:** High-intensity interval training, Self-selected recovery, Affect, Enjoyment, Mood, Cardiometabolic

## Abstract

**Introduction:**

High-Intensity Interval Training (HIIT) with self-selected recovery can promote positive emotional responses. This study aimed to verify the acute responses to HIIT with fixed recovery and to select high scorers on the state of affect, enjoyment, and mood in healthy young people.

**Methods:**

Nineteen participants took part in the study (19.0±1.0 years, 64.0±9.2 kg, 169.1±8.5, and 22.0±2.0 BMI). They performed 10 x 30 s (95% V_peak_) with 1 min recovery (HIIT_RecA_ - active - 40% V_peak_) and self-selected (HIIT_Rec-B_). Affect, enjoyment, and mood were assessed before and after HIIT (both conditions).

**Results:**

In both conditions, for time 1 and 2 were positive in positive (*p*=.170, EF: .102) and negative (*p*=.0.90, EF: .151) affect, pleasure (*p*=.010, EF: .315), and mood domains (tension: *p*<.001, EF: .673; depression: *p*=.015, EF: .286), anger: *p*=.033, EF: .230, and mental confusion: *p*<.001, EF: .451). In contrast, there was a reduction in the vigor domain and an increase in fatigue, with no differences in all emotional variables for condition and time*condition (*p*>.05).

**Conclusion:**

Thus, selected self-recovery may be a strategy in prescribing HIIT with possibilities of not interfering with the emotional aspects, researched here.

## 1 INTRODUCTION

High-Intensity Interval Training (HIIT) is an excellent strategy for physiological improvement [1, 2], either in terms of health [3] or in terms of performance [4, 5]. In health, HIIT has been widely reported to promote improvement in several indicators: cardiometabolic [6], cardiovascular [7], cardiorespiratory [8], psychological [9], and neurological [10]. As far as performance is concerned, HIIT is effective for improving movement economy [11], neuromuscular [12], cellular [13], maximal oxygen consumption (VO_2max_) [14, 15], and factors that influence the improvement of VO_2max_ [16]. All these functionalities act in an integrated way in the organism, allowing more efficiency and better physiological adaptations [17].

However, HIIT is capable of developing physiological adaptations [18, 19] responsible for different central responses [1] and peripheral [2] in individuals of different ages [20]. Additionally, some studies [21] on intervention with HIIT have shown interesting responses on psychological aspects. In this way, we can highlight that interventions with HIIT can offer significant results in behavioral evaluations of affect [21], enjoyment [22], and mood [23]. These behavioral variables were the focus of this research. Regarding affect and pleasure, HIIT proved essential for these responses, which are predictors of adherence to physical exercise [24]. Although studies cite combining high-intensity stimuli with adequate recovery periods for affective and pleasure responses [21], the mechanisms behind these results are still inconsistent [25].

In the mood, it is known that the type of intensity promotes optimal results in this behavior, and studies have shown that HIIT can be an essential strategy in this promotion [23]. Mood state is linked to cognitive function, especially task accomplishment [26]. In summary, studies with HIIT in psychological assessments are still scarce [27] because it is important to have more information to explain the mechanisms that influence the psychological responses to high-intensity exercise intervention [28]. In this sense, previous studies [10] presented evidence that HIIT positively affects the part of the prefrontal cortex, the region of the brain responsible for executive function, by increasing activation and oxygenation and thus making it more efficient. However, there is a need to control the intensity because it can interfere with the functional limit, resulting in mental fatigue, affecting task performance, and, consequently, mood [29]. However, HIIT is efficient in mood responses by providing stimuli in a controlled manner, even at a high intensity [10, 23]. In general, HIIT can be a positive intervention (in a planned way) in a training program aimed at improving health-related aspects [30, 31]. With regard to emotional state, we can suggest that high-intensity stimuli are effective in eliciting positive responses.

In prescription, another aspect that still needs more studies is related to the type of recovery between stimuli [30], as this is an important training variable, especially concerning HIIT [31]. Although some studies have already shown the importance of different types of recovery [32, 33], there is still a need to investigate the responses obtained by this important variable of HIIT prescription, especially considering psychological aspects [33, 34]. Thus, how one recovers between high-intensity stimuli is a question that needs further investigation because the type of recovery may be a determinant of psychological responses [33], thereby making the HIIT session more efficient [35] and attractive [36].

Active recovery [37] and passive recovery [38] are commonly used in interval training protocols and also serve as programmed break times, respecting physio-logical issues, mainlyioenergetic aspects [39]. However, one option that may also be a prescription strategy is the self-selected recovery [40]. In HIIT, because it is considered an activity that promotes optimal motivational level [41], the self-selected recovery can be a strategy to further increase the activity's pleasure [42]. In this way, it gets the individual to stay in the activity with efficient performance and thus tolerate high intensities [43]. However, even with these findings on self-selected recovery in interval training, there is still a large gap in this strategy. In addition, when it comes to HIIT, the demand for information on self-selected recovery is even greater, since this type of protocol requires efficient recovery/pauses between stimuli so that subsequent stimuli can be performed with optimum performance, minimizing physiological stress.

### 1.1 Present Study

Although some studies have investigated the influence of HIIT on affect, pleasure [21, 25], and mood [23], we have little information about the influence of the type of recovery in HIIT on these emotional states [44]. However, we know that HIIT can be a training option to promote positive psychological responses [45]. This way, active recovery with fixed time and selected high intensity can demonstrate distinct responses, even if positive in different magnitudes. Furthermore, we believe that the type of retrieval that occurs between stimuli may interfere with perceptual responses [33].

Studies have shown that HIIT is a strategy for promoting affect responses [46], enjoyment [44], and mood [23]. Therefore, the present study aimed to investigate the acute HIIT intervention with fixed recovery (HIIT_Rec-A_ 1 min. - active) and self-selected (HIIT_Rec-B_) on psychological behavior, using levels of affect, pleasure, and mood. Meaningful responses of HIIT with fixed recovery and self-selected recovery have already been identified [47, 48], but not on emotional aspects. Thus, analyses of the state of affect, pleasure, and mood are important because these variables can indicate important responses for the direction of training, emphasizing psychological analyses. In this sense, knowing more about these psychological variables is highly relevant to providing more tools for practical applicability, especially using HIIT. In addition, providing another recovery type option in HIIT (*e.g*., self-selected recovery) may be one more reason to make the method even more attractive and thus increase the chances of adherence to the activity. Finally, another hypothesis of the present study was that in HIIT_Rec-B_, the emotional levels (affect, pleasure, and mood) showed better [42].

## 2 METHODS

### 2.1 Participants

Sample size and power calculations were developed using G*Power (v.3.1.9.7) [49]. Considering the analysis to be performed on the primary outcomes, a between-within ANOVA-RM (2 [interventions] x 2 [time points]), anticipating a “large” effect size (*f* = 0.4), with an α = .05, a statistical power of (1 – β) = .95, the correlated dependent variables with a r = .50, and a violation of sphericity (ε) = .80, will require a total sample size of 18 individuals. The suggested effect size and remaining parameters were defined according to similar studies evaluating changes in affect, enjoyment, and mood during exercise protocols [24, 25, 46, 50].

The sample was composed of 19 participants (Table **1**), of whom 13 were men, and 6 were women (19±1.0 years; 64.0±9.2 kg; 169±8.5cm; 22.0±2 BMI), all healthy and active. All the participants selected were university students and were invited to take part in the study after a formal individualized notification. Inclusion criteria were as follows: Active exercisers with at least ≥ 6 months experience and a minimum weekly frequency of 3 times a week. As exclusion criteria, we considered the following: use of any pharmacological medication and/or ergogenic resources that could influence the investigation (*e.g*., those that could affect emotional state); and presentation of musculoskeletal disorders that could compromise the training protocol. All participants signed an informed consent form before the study intervention.

### 2.2 Design

**Fig. (1):**
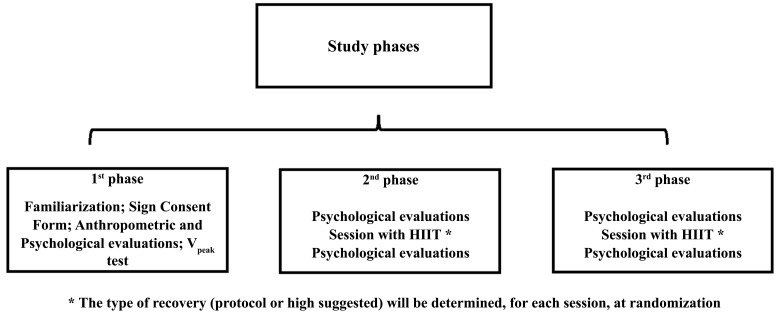
Experimental design.

For each visit, the present study was conducted for three laboratory sessions for approximately 30 minutes (Fig. **1**). The analyses were subdivided into each session, following the phases: the first phase: going to the laboratory for familiarization with the whole study, to sign the informed consent form, anthropometric evaluations (weight, height, and BMI), and performance of the maximal aerobic speed test (V_peak_) for determining training inten-sities. Second and third phases: going to the lab for the first and second intervention sessions. The intervention sessions were randomized to determine which protocol the individual would perform at each visit: HIIT_Rec-A_ (fixed recovery) or HIIT_Rec-B_ (self-selected recovery). Before and after the HIIT sessions, the subjects completed question-naires to evaluate affect, pleasure, and mood. The post-effort evaluation was started five minutes after the session's end to eliminate the effort's immediate influence on the responses, thus reducing the positive or negative bias on the emotional results. From the first to the second phase, the interval was 24 hours between sessions, and from the second to the third phase, the participants were 48 hours apart. All the data was collected by a single researcher to make it possible to have uniform evaluations among all the participants.

The participants' state of affect, enjoyment, and mood was investigated based on recent studies [21, 25, 47, 50]. The emotional evaluations were made through specific questionnaires for each behaviour based on studies related to the theme [23, 25, 51]. The evaluations were performed before and after the HIIT session for both conditions (HIIT_Rec-A_ and HIIT_Rec-B_).

### 2.3 Affect Evaluation

The positive and negative affect schedule will be used to assess effectiveness levels and psychometric properties [52]. The questionnaire has 20 items to be answered on a scale of 1 to 5, but we considered 5 for positive affect (items 1,10,15,17, and 19) and 5 for negative affect (items 2, 6, 7, 16, and 20), based on a study [53]. The sum of the positive and negative items was performed for the resolution. The higher the total value for positive affect and the lower the value for negative affect, the better the results related to this behavior.

### 2.4 Enjoyment Evaluation

For the evaluation of pleasure, the Physical Activity Pleasure and Feelings Scale (PACES) [54] was utilized. This questionnaire has 8 items related to the feeling of pleasure, answered on a scale of 1 to 7. All items are summed up, and the higher the total values, the better the pleasure index.

### 2.5 Mood Evaluation

For mood evaluation, the Brunel scale (Brums) will be used [55]. This questionnaire has 24 items subdivided into 6 domains on a scale of 0 to 4, being: tension (items 1, 13, 14 and 18), depression (items 5, 6, 12 and 16), anger (items 7, 11, 19 and 22), vigor (items 2, 15, 20 and 23), fatigue (items 4, 8, 10 and 21), and mental confusion (items 3, 9, 17 and 24). After summing up the responses for each item, the values for tension, depression, anger, fatigue, and confusion are expected to be decreased. Moreover, the domain values of vigor are maintained or increased.

### 2.6 Anthropometric Measures

The anthropometric evaluation was performed using the variables of weight (kg), height (m), and body mass index (BMI, kg/m^2^). For the identification of weight and height, a brand scale, TANITA^®^ (MC 580m), and a brand stadiometer SECA^®^ (AS 217) were used, respectively. Both were duly tested and calibrated. As for the BMI, it was obtained using the formula:

**Table:** 

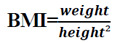

### 2.7 Peak Velocity Test (V_peak_)

The test for peak speed identification (V_peak_) was performed on a professional treadmill of the brand NORDIC TRACK^®^ (*T* 22.0). An incremental test was performed according to the Conconi protocol [56, 57] and in studies demonstrating the validity of_peak_ for intensity targeting [58-60]. The protocol was composed of an Initial Phase (warm-up): 5 min at a speed of 8.0 km/h and a sequence of static stretches with a resistance of 20 seconds for each exercise. Main phase (test): start at 8.0 km/h with a 1.0 km/h increment every one minute until voluntary exhaustion. Final phase (deceleration): 5 min at 5.0 km/h. The maximum speed reached was considered the one maintained for at least 50% of the determined time (1 min) for each increment.

### 2.8 Training Protocols

For the intervention, we will use a HIIT protocol performed on a treadmill, based on studies on the method [1, 61, 62]. The HIIT was conducted as follows: Initial Phase (warm-up): 10 min. (50% V_peak_). Main phase (stimulus/recovery): 10 x 30 sec (95% V_peak_) for 1 min active recovery (40% V_peak_) (HIIT_rec A_) or self-selected recovery (HIIT_rec B_). The self-selected recovery will be the convenient time (self-rated) for each individual to return to the next stimulus, the final phase (cool down): 5 min. (50% V_peak_).

### 2.9 Statistical Analysis

Means and standard deviations were calculated for all studied variables. Normality and homoscedasticity were verified with the Shapiro-Wilk (n < 50) and Levene's tests, respectively. Next, a within-between ANOVA 2x2 - (2 [interventions] x 2 [time points]) was conducted to examine differences between dependent variables. For all tests, the significance level to reject the null hypothesis was set at 5%. Sphericity assumptions were examined using Mauchly's test. When this assumption was not met, the Greenhouse-Geisser adjusted values and degrees of freedom were reported [63] and are indicated by the presence of decimal degrees of freedom. Bonferroni-adjusted post-hoc tests followed the repeated measures analyses to analyze pairwise comparisons. The η2p effect size was calculated, and the assumed reference values were as follows: “small” affect = .01, “medium” affect = .06, and “large” affect = .14 [64]. Statistical analyses were conducted in IBM SPSS Statistics version 27.

## 3 RESULTS

All the selected participants (n=19) completed all the stages of the study. At no time did they report any myoarticular discomfort or any other type of pain or injury. In the HIIT_Rec-B_ condition, the average time of the self-selected recovery time was 46.70±16.58 seconds. Table **2** shows the descriptive statistics for the variables studied. In general, the results in both conditions for times 1 and 2 were positive in the affect, pleasure, and mood domains of tension, depression, anger, and mental confusion. In contrast, there was a reduction in the vigor domain and increased fatigue.

In Table **3**, it is possible to observe that there are no differences in all emotional variables for condition and time*condition. However, a significant difference was observed in pleasure (*p*<.010), tension (*p*<.001), depression (*p*<.015), anger (*p*<.015), and mental confusion (*p*<.001) over time, and the observed affection fluctuates from small to medium-sized.

## 4 DISCUSSION

Although some studies already show positive results in affect [27], pleasure [21], and mood [44], information about the type of recovery of emotional aspects is still inconsistent [34], especially about affect, pleasure, and mood being assessed together and in front of exercise at high intensity. Regarding self-selected recovery, it becomes even scarcer [42]; therefore, the present study aimed to investigate the acute HIIT intervention with fixed recovery (HIITRec-A 1 min - active) and self-selected recovery (HIITRec-B) on psychological behavior, using levels of affect, enjoyment, and mood.

One of the initial hypotheses was that HIIT_Rec-B_ would promote better emotional responses (affect, enjoyment, and mood), as participants would have input into the methodological conduct of the session, and this, perhaps, would be a reason for better emotional responses. However, this theoretical assumption was not confirmed as there were no differences between the conditions. However, the other hypothesis would be that with the results, we could reinforce that the self-selected recovery could be one more strategy to manipulate this variable in sessions with HIIT. In this sense, self-selected recovery, as shown in other studies [34], is an excellent option for recovery following high-intensity stimuli.

### 4.1 Present Intervention and Responses of Affection, Pleasure, and Mood

Our findings showed, through descriptive statistics, positive responses for both conditions (in time: pre and post) on affect (positive and negative), enjoyment, and the mood domains: tension, depression, anger, and mental confusion. Additionally, in the mood domains, there was a decrease in vigor and an increase in fatigue, both negative responses. However, because the analysis focused on acute responses, participants were influenced by the effort performed within acceptable magnitudes. No significant differences were observed between time conditions for the emotional variables (*p* > .05). In contrast, significant differences were observed for pleasure and mood domains, including tension, depression, anger, and mental confusion, in both conditions when comparing pre- and post-intervention time points (*p* < .001).

These observed affections fluctuated between small and medium. Studies have shown positive responses after intervention with HIIT [27]. Alarcón-Gómez *et al*. [51] found optimal results after a 6-week HIIT intervention, and one of these results was an increase in enjoyment. However, most studies also relate the assessment of enjoyment, and improvements in the state of these variables are reinforced with the HIIT intervention [21].

After HIIT intervention with different recovery strategies, our findings, in part, reinforce those found in these cited studies. In mood, our findings were in line with those of Marques *et al*. [45], who also observed positive results in tension, depression, anger, and mental confusion and negative results in vigor and fatigue. In the present study, a significant difference was observed considering the time (pre and post) for the domains of tension, depression, anger, and mental confusion (*p*<.001) and not for vigor and fatigue (*p*>.05). Marques *et al*. [45] found significance (*p*<0.05) in the tension domains and for vigor, between groups.

It has already been emphasized that self-selected recovery [41] is important. In perceptual assessments (*e.g*., ratings of perceived exertion) with HIIT, studies also reinforce that self-selected recovery can be considered an option in the prescription for these assessments [36, 43], demonstrating that this type of recovery can be a tool for psychological influence. However, the present study aimed to investigate the influence of self-selected active recovery in HIIT on affect, enjoyment, and mood states compared to traditional active recovery with a fixed time (the 60s).

Santos *et al*. [42] used self-selected recovery (passive) retrieval between stimuli and observed no significant differences in affect but demonstrated that this type of retrieval strategy might be relevant in HIIT prescriptions. Although these studies demonstrate the potential in the selected high recovery, we have a considerable restriction of studies using this recovery strategy in HIIT assessing affect, enjoyment, and mood. In the present study, in the HIIT_RecB_ condition, the average time of the selected high recovery was 46.70±16.58 seconds. Not much different from the time set (the 60s) in the HIIT_RecA_ condition, demonstrating that the participants could apply an adequate time for performing the recovery.

The self-selected recovery may be significant not only for physiological responses [43, 49] but also for psychological ones [36, 42]. However, more studies with evaluations on affect, pleasure, and mood are still needed. The fact that the individual participates in the methodological conduction of the session (*e.g*., self-selected recovery) may be a strategy to promote affect and pleasure, which are also related to adherence to the activity [36, 65]. Additionally, it is agreed that strategies such as self-selected exercises play a crucial role in positive affective responses [66].

Our findings reinforce the idea that self-selected recovery can be an option within the HIIT configuration and elicit positive affective responses, consistent withhe Dual-Mode Theory proposed by Ekkekakis [67]. This theory agrees on the importance of pleasure in prescribing and monitoring activity, assuming that hedonic factors are an essential part of the framework surrounding the psychological responses to activity [47]. HIIT can be an exercise option with broad adherence capacity [42] because it provides optimal responses in affection and enjoyment [47, 68, 69], these being indicators of permanence in the activity [70].

HIIT is an efficient method for physiological and psychological responses [70], which makes this activity important and considered in the prescription of aerobic activities. Finally, the type of recovery between stimuli is a crucial variable in programming the HIIT session [30, 71, 72]. This way, we can suggest that self-selected recovery is another prescription option, supported by the studies [40, 42, 47, 73].

### 4.2 Possible Influencing Mechanisms

The mechanisms behind the affect, enjoyment, and mood responses arising from HIIT intervention (regardless of the type of recovery) are complex, and studies report inconsistency on this part [25]. Affective responses are related to conscious mental processing [74]. In neural terms, studies suggest that the frontal lobe plays a crucial role in affective modulation and that these emotional changes can be positive in aerobic activities [75]. In this sense, we can reinforce that HIIT, despite being composed of anaerobic stimuli [2, 76], is an activity that has aerobic predominance [1, 61, 77] and can be an excellent intervention strategy.

Furthermore, regarding the frontal lobe, asymmetric activation of this brain region appears to indicate effective responses [75, 78]. About enjoyment, it is a subjective hedonic variable linked to neuropsychological components that require biological brain resources [79]. From a neurochemical point of view, studies point to dopamine as the primary neurotransmitter related to the state of affection and enjoyment [80]. In mood, cortical connectivity seems to trigger positive responses in this behavioural state [81, 82]. Another issue is that mood is linked to cognitive function, mainly when accomplishing tasks [26].

In this sense, previous studies [10] presented evidence that HIIT positively affects the part of the prefrontal cortex, the region of the brain responsible for executive function, by increasing activation and oxygenation and thus making it more efficient. However, there is a need to control the intensity because it can interfere with the functional limit, resulting in mental fatigue, affecting task performance and consequently the mood [29], generating a negative consequence in the state of affection and enjoyment [36]. However, the mechanisms linked to improving these emotional aspects of high-intensity exercise are still unclear [67, 81].

### 4.3 Limitations, Practical Applications, and Future Studies

The study has some limitations, one of which is the scarcity of explanations about the mechanisms behind the affect, pleasure, and mood responses to high-intensity exercise. Regarding HIIT intervention in psychological evaluations, the studies are limited to explaining the mechanisms, perhaps because of the same difficulty we had. However, there is a relevant essential to have more information to explain the mechanisms that influence the psychological responses resulting from high-intensity exercise intervention, especially the variables analyzed here [28]. Another limitation is that we cannot extrapolate our findings chronologically because we performed a critical analysis. Given that our sample (n=19) followed the requirements of the sample calculation (n=18), we cannot extrapolate the findings to the entire population of young people. However, we can suggest that our findings are important parameters to consider.

Studies that used HIIT to analyze these emotional states over extended periods obtained positive results [21, 47, 50], which reinforce the idea that this type of intervention is an option for improving psychological states. Additionally, we used a recovery strategy, a fixed time, and a selected high. In interval training, recovery between stimuli is performed either actively or passively, and both are important in the session [30]. In general, these strategies are carried out within a fixed time. However, studies already advocate the self-selected recovery model as another application form [42, 83]. Our findings add to this argument that recovery within the time chosen by the practitioner may play an essential role in HIIT sessions, especially in emotional responses [83].

In this way, we can point out that this variation of recovery used adds more attractiveness to HIIT and can make this activity more motivating and consequently be a reason for adherence to the practice [41]. We suggest other studies with this theme, with more variations of the type of recovery (for example, active *versus* passive), other protocols, and for a longer analysis time. Thus, it will be possible to further enrich HIIT using a self-selected recovery. Finally, the present study has proven to be very useful for practical applicability. We used a very accessible intervention methodology, and in the same way, we evaluated the responses of the interventions with easily applicable and highly valid instruments. Thus, the findings of this research managed to achieve a high level of utilization and contribution to the prescription of physical training.

## CONCLUSION

The present study used HIIT interventions using two active recovery conditions between stimuli: one with a fixed time (the 60s) and the self-selected recovery time. To analyze emotional variables, such as affect, pleasure, and mood, we found that both conditions helped to achieve positive responses. With this, we can reinforce, along with other studies already done, that in addition to the traditional ways of recovering between stimuli in HIIT, the high selected recovery can also be a strategy, and with that, we can have one more methodological tool to apply in training with this method.

## Figures and Tables

**Table 1:** Characteristics of the sample in general.

Anthropometric Variables	Sample	
	Mean	Sd
Age (years)	19.0	1.0
Weight (kg)	64.0	9.2
Height (cm)	169.1	8.5
BMI (kg/m^2^)	22.0	2.0
Mechanical variables (km/h)	Sample	
	Mean	Sd
V_peak_ test (100%)	16.5	2.8
V_peak_ rec (40%)	6.6	1.4
V_peak_ W/C (50%)	8.2	1.4

**Note:** BMI: body mass index; Vpeak speed test; Vpeak rec: speed recovery; Vpeak W/C: speed warm and cool down.

**Table 2:** Descriptive statistics for affect, enjoyment, and mood.

**Emotions Variables**	**M_preC1_**	**M_posC1_**	**SD_preC1_**	**SD_posC1_**	**M_preC2_**	**M_posC2_**	**SD_preC2_**	**SD_posC2_**
Positive Affect	3.30	3.43	.66	.77	3.32	3.49	.64	.80
Negative Affect	1.13	1.06	.28	.13	1.30	1.20	.33	.43
Enjoyment	4.74	5.15	.62	.95	5.05	5.24	.87	1.13
Tension	2.26	2.07	.58	.838	2.95	2.44	.79	.75
Depression	1.10	.68	946	.32	1.32	.37	.95	.16
Anger	.68	.32	1.56	1.16	1.00	.11	1.56	.32
Vigor	7.26	6.68	2.42	2.89	7.37	6.95	2.63	3.10
Fatigue	3.21	3.74	2.29	3.63	4.32	4.84	2.92	3.74
Mental confusion	1.11	.32	1.99	.67	1.26	.32	1.14	.75

**Note**: M_preC1_= mean pre-test condition 1; M_posC1_= mean pos-test condition 1; SD_preC1_= standard deviation pre-test condition 1; SD_posC1=_ standard deviation pos-test condition 1; M_preC2_= mean pre-test condition 2; M_posC1_= mean pos-test condition 2; SD_preC2_= standard deviation pre-test condition 2; SD_posC2_= standard deviation pos-test condition 2.

**Table 3:** Differences in all emotional variables for time, condition, and time*condition.

Emotions Variables	Mean Square	F	df1	df2	*p*	η^2^_p_	Pairwise Comparisons
**Positive Affect**							
Time	10.316	2.048	1	18	.170	.102	ns
Condition	.842	.050	1	18	.826	.003	ns
Time*Condition	.842	.057	1	18	.814	.003	ns
**Negative Affect**							
Time	11.066	3.203	1	18	.090	.151	ns
Condition	3.803	3.923	1	18	.063	.179	ns
Time*Condition	.474	.073	1	18	.790	.004	ns
**Pleasure**							
Time	108.961	8.265	1	18	.010	.315	1 ≠ 2
Condition	48.961	.580	1	18	.456	.031	ns
Time*Condition	57.316	1.358	1	18	.259	.070	ns
**Tension**							
Time	70.118	36.978	1	18	<.001	.673	1 ≠ 2
Condition	3.803	.873	1	18	.673	.046	ns
Time*Condition	4.263	.561	1	18	.463	.030	ns
**Depression**							
Time	8.224	7.212	1	18	.015	.286	1 ≠ 2
Condition	2.224	.860	1	18	.366	.046	ns
Time*Condition	6.368	1.670	1	18	.213	.085	ns
**Anger**							
Time	7.579	5.366	1	18	.033	.230	1 ≠ 2
Condition	.053	.029	1	18	.867	.002	ns
Time*Condition	5.263	.888	1	18	.359	.047	ns
**Vigor**							
Time	4.750	.966	1	18	.339	.003	ns
Condition	.645	.059	1	18	.811	.001	ns
Time*Condition	.474	.082	1	18	.778	.005	ns
**Fatigue**							
Time	5.263	2.337	1	18	.419	.037	ns
Condition	23.211	.683	1	18	.144	.115	ns
Time*Condition	.001	.022	1	18	.998	.001	ns
**Mental confusion**							
Time	14.329	14.805	1	18	.001	.451	1 ≠ 2
Condition	.118	.049	1	18	.828	.003	ns
Time*Condition	.474	.103	1	18	.752	.006	ns

**Note:** F = test results; df1 = degrees of freedom of the six conditions; df2 = degrees of freedom of error; p = significance; η^2^_p_ = partial eta-square; ns= no differences detected.

## Data Availability

All data generated or analyzed during this study are included in this published article.

## LIST OF ABBREVIATIONS

HIIT: High-Intensity Interval Training
VO_2max_: Maximal Oxygen Consumption
V_peak_: Peak Velocity
